# A Study on the Optimal Magnetic Beam Forming of Coil Arrays for Long Distance Wireless Power Transmission

**DOI:** 10.3390/s23115312

**Published:** 2023-06-03

**Authors:** Myeong-Jun Oh, Patrick Danuor, Young-Bae Jung

**Affiliations:** Department of Electronics Engineering, Hanbat National University, Daejeon 34158, Republic of Korea

**Keywords:** beam steering, multiple-input multiple-output (MIMO), multiple transmitters, phase control, power transfer efficiency (PTE), wireless power transfer (WPT)

## Abstract

Multiple-input multiple-output (MIMO) wireless power transfer (WPT) technology which employs multiple transmitter (TX) coils to simultaneously couple power to the receiver (RX) coil has proved to be an effective technique to enhance power transfer efficiency (PTE). Conventional MIMO-WPT systems rely on the phase-calculation method based on the phased-array beam steering concept to constructively combine the magnetic fields induced by the multiple TX coils at the RX coil. However, increasing the number and distance of the TX coils in an attempt to enhance the PTE tends to deteriorate the received signal at the RX coil. In this paper, a phase-calculation method is presented that enhances the PTE of the MIMO-WPT system. The proposed phase-calculation method considers the coupling between the coils and applies the phase and amplitude to calculate the coil control data. From the experimental results, the transfer efficiency is enhanced as a result of the transmission coefficient improvement from a minimum of 2 dB to a maximum of 10 dB for the proposed method as compared to the conventional one. By implementing the proposed phase-control MIMO-WPT, high-efficiency wireless charging is realizable wherever electronic devices are located in a specific space.

## 1. Introduction

Wireless power transfer (WPT) technology continues to witness a tremendous growth as it proves to be a relatively effective mode of power supply for a wide range of applications including electric vehicles, medical implants and consumer electronics [1,2]. The power transfer efficiency (PTE) of WPT systems, however, greatly depends on how well power can be coupled from the transmitter (TX) coil to the receiver (RX) coil [3].

WPT via inductive coupling achieves a higher transfer efficiency providing the TX and RX coils are placed in close proximity and are perfectly aligned with each other. The PTE, however, deteriorates drastically as the TX and RX coil distance increases [4]. Thus, WPT via resonant inductive coupling has been proposed in several studies [5]. WPT via resonant inductive coupling can exchange power efficiently with much larger transfer distance compared with the conventional WPT via inductive coupling. However, for WPT via resonant inductive coupling, the TX–RX coil distance must be optimized below a threshold value to prevent over-coupling, which deteriorates the PTE [6].

**Figure 1:**
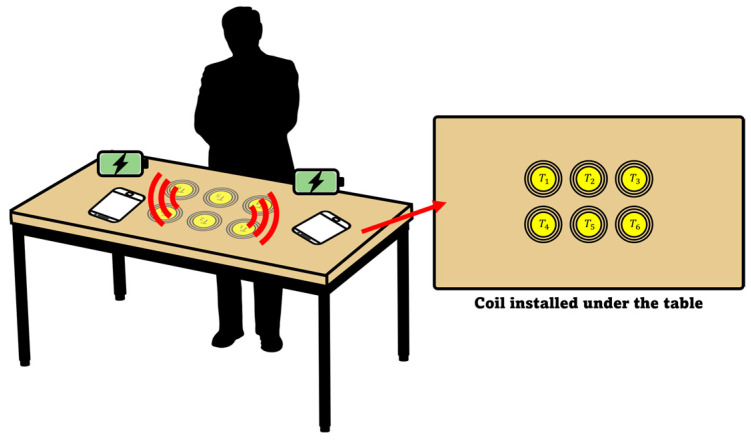
Illustration of the MIMO-WPT system with multiple TX coils installed underneath the table.

Recently, much attention has been focused on finding alternative means to provide a higher degree of freedom and safety to improve the user experience of WPT. The use of multi-coil configurations such as the multiple-input multiple-output (MIMO) WPT systems which consist of multiple TX/RX coils, or both, have been presented in several studies [7,8]. The MIMO-WPT system usually consists of multiple TX coils to simultaneously transmit magnetic energy to a single RX coil, in order to attain the benefits of added gain and diversity effects [9]. The magnetic field-based MIMO wireless charging method shows higher transmission efficiency because it can transmit high amounts of power by synthesizing magnetic fields generated in individual transmission coils. Figure 1 illustrates the MIMO-WPT system with the TX coils installed underneath the table that delivers the power to charge a device placed at an arbitrary position on the table.

For efficient long-distance wireless power transmission, a number of wireless charging solutions have been implemented using RF, infrared rays, ultrasonic waves, etc. Ossia’s Cota^®^ demonstrated the use of RF to provide long-distance wireless power via a device consisting of hundreds of omni-directional antennas [10]. Moreover, Energous Corp. has implemented a long-distance wireless power transmission using its RF-based WattUP^®^ technology that can charge multiple devices at the same time from a distance [11].

Furthermore, an ultrasonic wireless charging device named uBeam transmits a signal using a high ultrasonic band frequency of 45 kHz to 75 kHz. The ultrasonic transmitter is configured similarly to the electrical principles of the phased-array antenna, and forms an ultrasonic beam synthesized in free space through thousands of small speakers [12]. Additionally, a magnetic MIMO (MagMIMO) system has been developed by scientists at MIT, which can wirelessly charge cell phones and portable devices placed about 50 cm away using multiple TX coils [13].

Most of the literature studies presented above for long-distance wireless power transfer employ the conventional phase-calculation method based on the phased array concept to transmit power efficiently from multiple TX coils to a single RX coil.

In this study, we show that for the conventional phase-calculation method of the MIMO-WPT system, as the number and distance of the TX coils increases in a bid to increase the PTE, the synthesized magnetic field beam deteriorates, with the formation of multi-lobes. We further propose a new method to calculate the phase applied to the individual TX coils which incorporates the coupling coefficient between the TX coils, in order to transmit optimal power to the RX coil placed at an arbitrary position by the MIMO-WPT wireless charging method depicted in Figure 1.

## 2. MIMO-WPT System Modelling and Analysis

**Figure 2:**
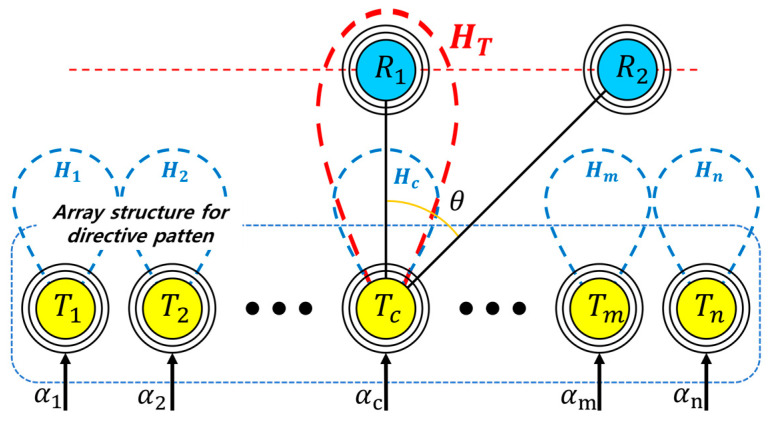
Schematic diagram of the MIMO-WPT system.

Figure 2 shows the schematic diagram of a MIMO-WPT system composed of *N* number of TX coils (i.e., *T*_1_, *T*_2_,…, *T_n_*) and two RX coils (i.e., R_1_ and R_2_) placed at arbitrary positions above the TX coils. Assuming that all the coils have the same physical structure and that all TX coils are fed individually with the same current, it can be considered that the amount of magnetic energy (*H*) generated by the individual coils are the same. In the case where the phase of the current applied to the individual coils is the same, the magnetic fields (*H*_1_, *H*_2_, …, *H_n_*) generated from the individual coils are synthesized in an arbitrary direction (i.e., *θ* = 0⁰) to generate a summation of the magnetic field (*H_T_*) as illustrated in Figure 2. Thus, higher power may be transmitted compared to the case in which a single TX coil is used. When the RX coil moves horizontally by an angle of (*θ*) with respect to the center of the array, the phase of the individual TX coils must be controlled to optimize the magnetic energy for each RX coil position. Therefore, assuming that the position of the RX coil moves freely, the phase-control method applied at TX coils can be a key function for the efficient operation of the MIMO-WPT system.

### 2.1. Analysis of MIMO-WPT Using the Conventional Phase-Calculation Method Based on the Phased-Array Concept

**Figure 3:**
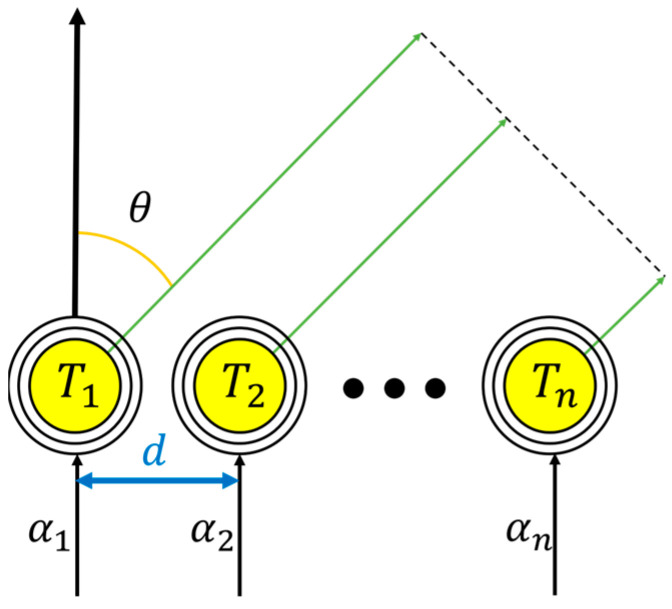
Illustration of the beam-steering concept of the phased-array antenna with coils implemented.

From the MIMO-WPT system illustrated in Figure 2, the concept of the beam-steering control employed for the phased-array antenna technology can be employed to determine the phases of the individual TX coils in order to transmit optimum magnetic energy to the RX coil. Figure 3 illustrates the application of the phased-array beam-steering control method to the MIMO-WPT system, where coils are used in place of antenna elements.

From the antenna array concept, the total radiated field (*E_ia_*) from an isotropic linear array of *N* number of elements can be represented as [14]

(1) $$E_{ia}=E_{ie}\times \sum_{n=1}^{N}A_{n}e^{j\alpha_{n}}$$

where *A_n_* and *α_n_* represent the amplitude and relative phases of the isotropic elements, respectively, and *E_ie_* is the radiation from an isotropic element. The second term in (1) is termed as the array factor (*AF*). The antenna array factor is the weight of the array antenna with periodic element spacing and represents the array response. Assuming that the spacing between the elements (*d*) is fixed and all the elements have identical amplitudes (i.e., an amplitude of 1), then the *AF* can be represented as

(2) $$AF=\sum_{n=1}^{N}e^{j(n-1)(\beta d\sin\theta +\alpha_{0})}=\sum_{n=1}^{N}e^{j(n-1)\psi }$$

(3) $$\psi =\beta d\sin\theta +\alpha_{0}$$

where *ψ* and *β* represent the phase difference between two adjacent elements and phase constant, respectively. In the conventional MIMO-WPT system, the *AF* is employed to derive the phase-shifts (*α*) between the TX coils, which are each separated by distance (*d*) to transmit optimum magnetic energy to the RX coil.

### 2.2. MIMO-WPT Based on the Proposed Phase-Calculation Method

In this study, a new phase-control method applied to the coils is presented to improve the transmission efficiency of the MIMO-WPT system. In the previously used phase-control method, the phases between each TX coil were determined and used to obtain the desired synthesized magnetic fields at the receiver. However, due to the close proximity of the coils, magnetic coupling exists between the TX coils and the TX–RX coils which results in reflected mutual impedances at the circuits of each of the TX and RX coils. Hence, a phase-shift due to the coupling from each circuit is introduced. Therefore, the phase of the input voltages of the coils has to be adjusted to align the currents with the same phase.

**Figure 4:**
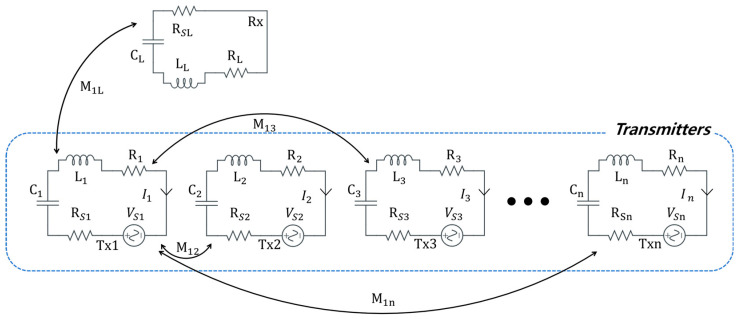
Circuit diagram of MIMO-WPT system with *N* number of transmitters and one receiver.

Figure 4 shows a circuit diagram composed of *N* number of TXs and one RX coil and depicts the mutual coupling (*M_mn_*) between the TX–TX and TX–RX coils. Each coil has capacitance (*C_n_*), inductance (*L_n_*) and resistance (*R_n_*). Each TX coil is supplied with a source voltage (*V_sn_*) and current (*I_n_*).

Based on Kirchhoff’s voltage Law (KVL), the impedance (*Z_n_*) for each TX coil can be represented in (4) as

(4) $$Z_{1\;\;}=R_{1}+j\omega L_{1}+1/j\omega C_{1}\;Z_{2\;\;}=R_{2}+j\omega L_{2}+1/j\omega C_{2}\;\vdots \;Z_{n\;\;}=R_{n}+j\omega L_{n}+1/j\omega C_{n}$$

The mutual inductances (*M_mn_*) are related to the coupling coefficients (*K_mn_*) by (5).

(5) $$M_{n,m}=k_{n,m}\sqrt{L_{n}L_{m}}$$

Based on the coupling coefficients between the coils, the phase and amplitude can be derived using the expressions in (6), (7) and (8) [15]. The phase and amplitude using the coupling coefficient between individual coils could be obtained mathematically by finding the coupling relationship between two coils using (6) and calculating the coupling between multiple coils using (7) and (8).

(6) $$V_{S1}=I_{1}Z_{1}-j\omega I_{2}M_{12}$$

(7) $$0\;=I_{2}Z_{2}-j\omega I_{1}M_{12}$$

(8) $$I_{2}=\frac{V_{S1}j\omega M_{12}}{Z_{1}Z_{2}+{\left(\omega M_{12}\right)}^{2}}$$

The voltage (*V_sn_*) and current (*I_n_*) expressions for the nth coil is given in (9) and (10) as

(9) $$V_{Sn}=I_{n}Z_{n}-j\omega I_{1}M_{1n}-j\omega I_{2}M_{2n}-j\omega I_{3}M_{3n}-\cdots -j\omega I_{n}M_{nL}$$

(10) $$I_{n}=\frac{V_{S1}+j\omega I_{1}M_{1n}+j\omega I_{2}M_{2n}+j\omega I_{3}M_{3n}+\cdots +j\omega I_{n}M_{nL}}{Z_{n}}$$

Using the phase data of the currents derived from (10), the phase applied to each TX coil ($\theta_{11}$), the phase of the current at the RX coil (*θ*_11_), and the phase of the current due to other TX coils ($\theta_{21}$*,* $\theta_{31}$*,* ⋯ *,* $\theta_{n1}$) can be obtained. The total phase at each of the TX coils is the sum of the phase shift due to the coupling with the RX coil, and the coupling due to the other TX coils. Therefore, the phase adjustment ($\theta_{n}^{T}$) and amplitude adjustment ($A_{n}^{T}$) at the *n*th TX coil can be expressed in (11) and (12) as

(11) $$\theta_{n}^{T}=\overset{L}{\underset{k=1}{\sum }}\theta_{kn},\;\forall i,\;i=1,\cdots ,n,L$$

(12) $$A_{n}^{T}=\overset{L}{\underset{k=1}{\sum }}A_{kn},\;\forall i,\;i=1,\cdots ,n,L$$

## 3. Experimental Results and Analysis

**Figure 5:**
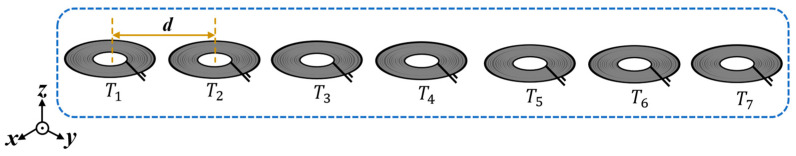
Illustration of the experimental set up for MIMO-WPT.

In this section, the effectiveness of MIMO-WPT based on the beam-steering phase-calculation method, and the MIMO-WPT based on the proposed phase-calculation method is verified and compared using ANSYS Maxwell electromagnetics software [16]. In the simulation set-up, the MIMO-WPT system illustrated in Figure 5 is used for different *n*-TXs (i.e., three-TXs, five-TXs and seven-TXs) configurations. The distance between the TX coils (*d*) is kept at 50 cm while the phase steps (*ψ*) of the TX coils are varied from *ψ* = 0°, 30°, 60° and 90°.

### 3.1. Experimental Results for MIMO-WPT Based on the Conventional Beam-Steering Phase-Control Method

Table 1, Table 2 and Table 3 show the distribution of the magnetic field (*H*) with varying phase steps (*ψ*) for three-TXs, four-TXs and five-TXs coil configurations. The ‘Azimuth’ and ‘Elevation’ columns of Table 1, Table 2 and Table 3 represent the cross-section of the simulated magnetic field intensity from the TX coils measured with respect to the *xy*- and *yz*-planes, respectively, as shown in Figure 5.

From the ‘Elevation’ results in Table 1, it can be seen that the synthesized magnetic field from the three TX coils realize a beam tilt in the left direction as *ψ* increases from 0° to 60°. However, when *ψ* = 90°, a beam split is observed with a beam tilt in the right direction.

Furthermore, from the results of Table 2, as *ψ* changes from 0° to 30°, the beam tilts significantly towards the left side compared to using the three-TX coil configuration in Table 1. However, at *ψ* = 60°, the radiated beam pattern deteriorates and tilts towards the right side resulting in a beam split. At *ψ* = 90° the beam splits into two different beam directions. It can be further realized that the magnetic field beam starts to deteriorate at *ψ* = 30° in Table 2 for the five-TX coil implementation compared to that of Table 1, which starts to deteriorate at *ψ* = 60°.

In Table 3, the simulated magnetic field (*H*) results are given for different *ψ* when seven TX coils are used. It can be observed that the magnetic beam pattern begins to deteriorate as *ψ* increase from *ψ* = 30° to 90°. Compared to the case of three-TXs and five-TXs in Table 1 and Table 2, a significant beam split is noticed for increasing *ψ* using seven-TXs.

**Figure 6:**
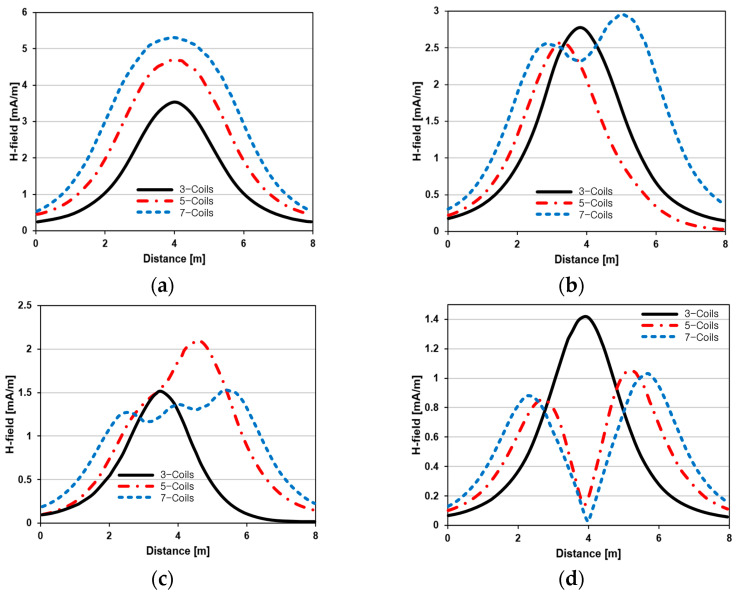
Beam-steering directions for different TX coil configurations: (**a**) tilt angle = 0°, (**b**) tilt angle = 30°, (**c**) tilt angle = 60° and (**d**) tilt angle = 90°.

The results in Table 1, Table 2 and Table 3 were compared according to the number of TX coils and the beam steering angle and are given in Figure 6. Figure 6 shows the change in the beam steering angle when the interval between coils (*d*) is set to 50 cm for the different *n*-TX of coil configurations. When *ψ* = 0°, it can be seen that the transmission efficiency increases as the number of TX coils increases. For the case of to *ψ* = 30°, multiple lobes are formed for the seven-TXs, and the beam moves towards the left side relative to the three-TXs and five-TXs. For *ψ* = 60°, the multi-lobes generated for the case of seven-TXs becomes more significant, and when three-TXs are used, the beam tilts more towards the left side compared to *ψ* = 30°. For *ψ* = 60° with the five-TX configuration, the beam tilts towards the right side. 

As *ψ* increased to 90°, multiple lobes were generated even for five- and seven-TX coils. However, for three-TX coils, the beam tilted towards the right side. As a result, it may be seen that as the coil arrangement increases, the beam steering angle is distorted, and multiple lobes are generated for the conventional phase-calculation method of the MIMO-WPT system.

**Figure 7:**
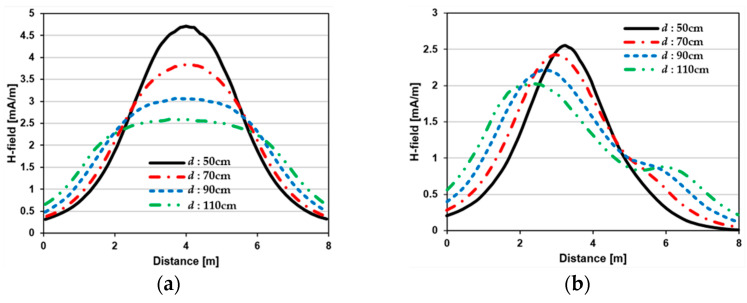

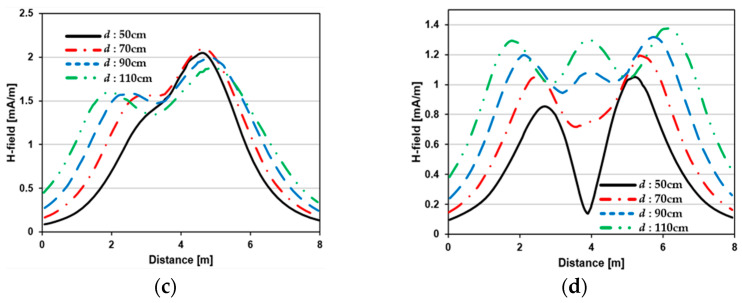
Beam steering direction with changing TX–TX coil distance (*d*) for the five-TX coil configuration: (**a**) tilt angle = 0°; (**b**) tilt angle = 30°; (**c**) tilt angle = 60°; (**d**) tilt angle = 90°.

Figure 7 shows the effect of varying the TX–TX coil distance (*d*) for different beam steering angles for the MIMO-WPT with the five-TX coil configuration. When tilt angle is 0°, the synthesized magnetic field beam decreases as the TX–TX distance increases. Additionally, it can be seen that when *ψ* = 30°, multiple lobe characteristics occur as *d* increases, and the beam steering direction changes towards the left side. When *ψ* = 60°, the multi-lobe characteristic becomes apparent as the distance increases. For *ψ* = 90°, the multi-lobe characteristics become more severe, resulting in two to three beam splits.

### 3.2. Experimental Results for MIMO-WPT Based on the Proposed Phase-Calculation Method

**Figure 8:**
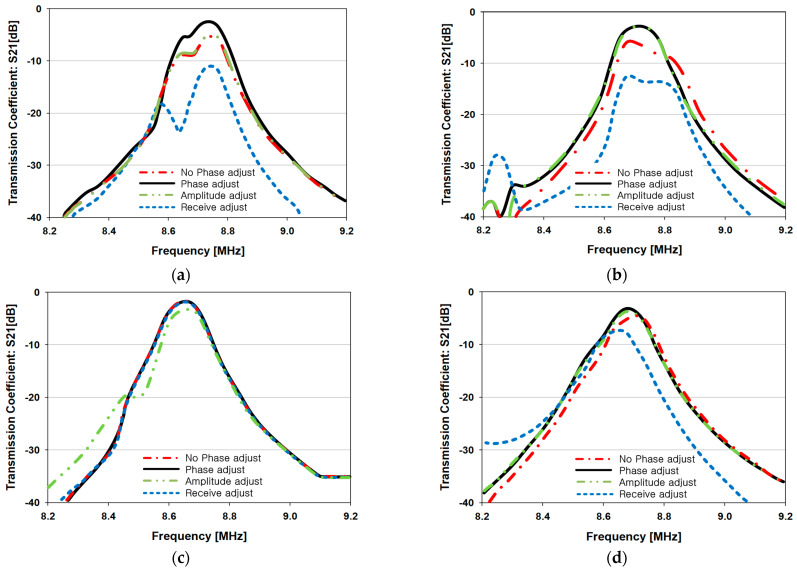

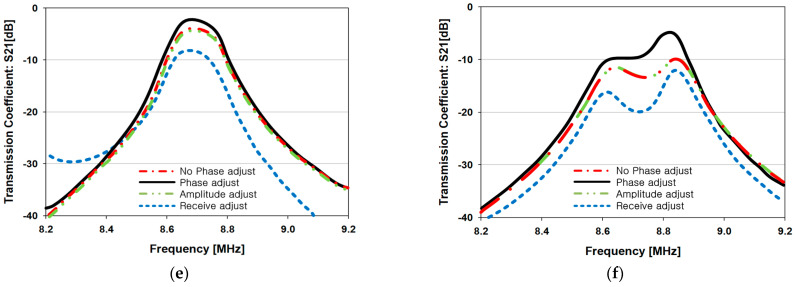
Comparison of different phase-control methods according to the beam steering angle for the five-TX coil configuration: (**a**) tilt angle = 0°; (**b**) tilt angle = 14°; (**c**) tilt angle = 26.5°; (**d**) tilt angle = 37.0°; (**e**) tilt angle = 51.0°; (**f**) tilt angle = 60°.

The simulation results for different MIMO-WPT phase-calculation methods using the five-TX coil configuration are given in Figure 8. In the results ‘No Phase adjust’, ‘Phase adjust’, ‘Amplitude adjust’, and ‘Receive adjust’ the phase-calculation method for each beam tilt angle are presented. ‘No Phase adjust’ represents the case where the coupling coefficient is not considered. ‘Phase adjust’ is the case where the proposed phase coefficient using (11) is used. For ‘Amplitude adjust’, the amplitude coefficient is applied using (12), and for ‘Receive adjust’, the coupling coefficient between each TX and RX coil is used.

Figure 8a shows the results for the case where the beam tilt angle is 0°, which shows that ‘Phase adjust’ has the highest transmission coefficient (S_21_). ‘Amplitude adjust’ and ‘No phase adjust’ are about 3 dB lower than ‘Phase adjust’, and ‘Receive adjust’ is about 8 dB lower than ‘Phase adjust’.

Figure 8b shows that the two methods, ‘Phase adjust’ and ‘Amplitude adjust’, have the highest transmission coefficients for the tilt angle measured at 14°. The results of ‘No phase adjust’ and ‘Receive adjust’ show about 3 dB and 8 dB lower, respectively, compared to the phase adjust and amplitude adjust. At a beam tilt of 26.5°, all the phase-calculation methods show similar performance as shown in Figure 8c.

In Figure 8d, for a beam tilt angle of 37°, the ‘Phase adjust’, ‘Amplitude adjust’ and ‘No phase adjust’ show similar results, however ‘Receive adjust’ shows a lower transmission coefficient of about 3 to 5 dB, comparatively. For a beam tilt of 51° as shown in Figure 8e, the ‘Phase adjust’ method shows the highest transmission coefficient, with the ‘Amplitude adjust’ and ‘No phase adjust’ methods showing similar results. However, the ‘Receive adjust’ method shows a transmission coefficient about 6 dB lower than ‘Phase adjust’. Moreover, in Figure 8f, the formation of multiple lobes is noticed for a beam tilt of 60°.

From the results of Figure 8, it can be confirmed that the ‘Phase adjust’ which is based on (11) exhibits the highest transmission coefficient compared to the other phase-calculation methods. Furthermore, it can be seen that ‘Amplitude adjust’ and ‘No phase adjust’ exhibit similar performance, indicating that ‘Amplitude adjust’ using (12) does not have a significant impact on transmission efficiency of the MIMO-WPT system. Since the ‘Receive adjust’ method shows the lowest transmission coefficient, it can be concluded that this method is not an efficient phase-calculation method to improve the PTE of the MIMO-WPT system.

The transfer efficiency (*η*) at the RX coil is related to the transmission coefficient (S_21_), and can be determined by [17]

(13) $$\eta \;=\;{\left\lfloor s_{21}\right\rfloor }^{2}\times 100\%$$

## 4. Conclusion

In this paper, different phase-control methods for improving the transfer efficiency of a MIMO-WPT system were presented. From these studies, it was realized that the beam pattern splits and deteriorates when the number and distance between the TX coils increases for the conventional phase-control method based on the beam-steering concept of the phased-array antenna. Additionally, multiple lobes appear for higher steering angles in the conventional phase-calculation method. Hence, a MIMO-WPT phase-control method is presented which incorporates the coupling between the coils in the phase-calculation method. The transmission efficiency is improved based on the results of the transmission coefficient, which shows an improvement from 2 dB to 10 dB compared to the conventional method.

## Figures and Tables

**Table 1:** Simulated magnetic field distribution of the beam-steering MIMO-WPT implemented with three-TX coils.

Phase Step (*ψ*)	Azimuth (*xy*-Plane)	Elevation (*yz*-Plane)
0°	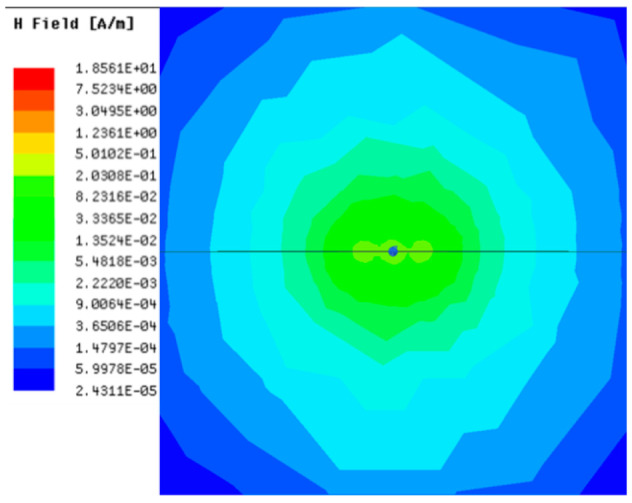	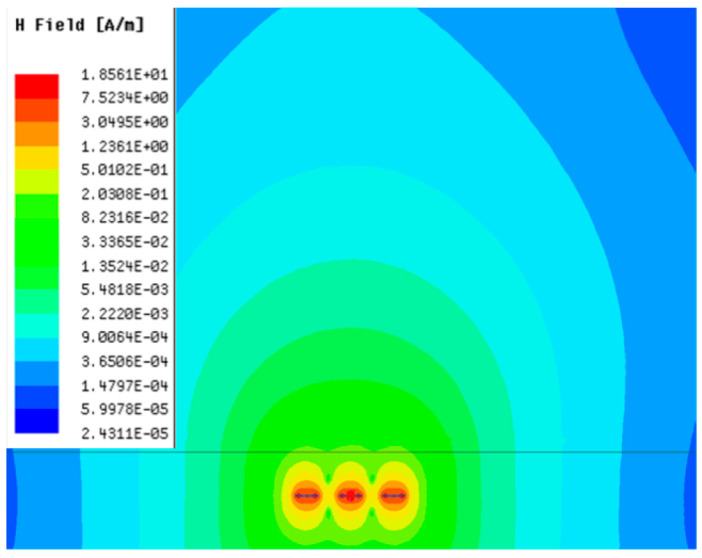
30°	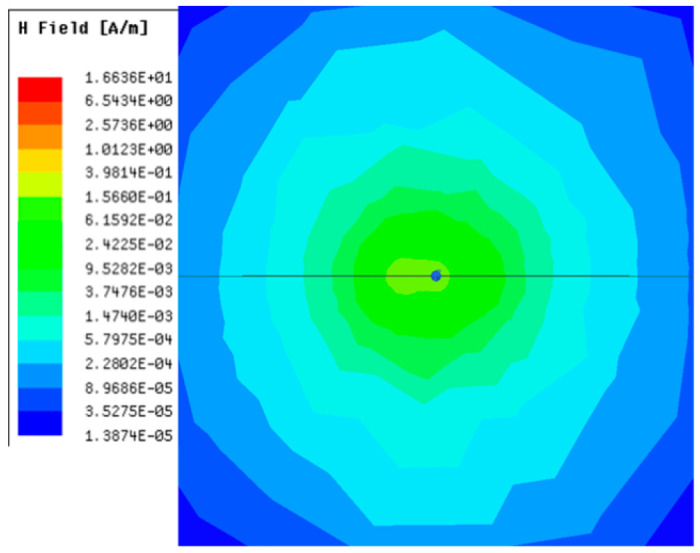	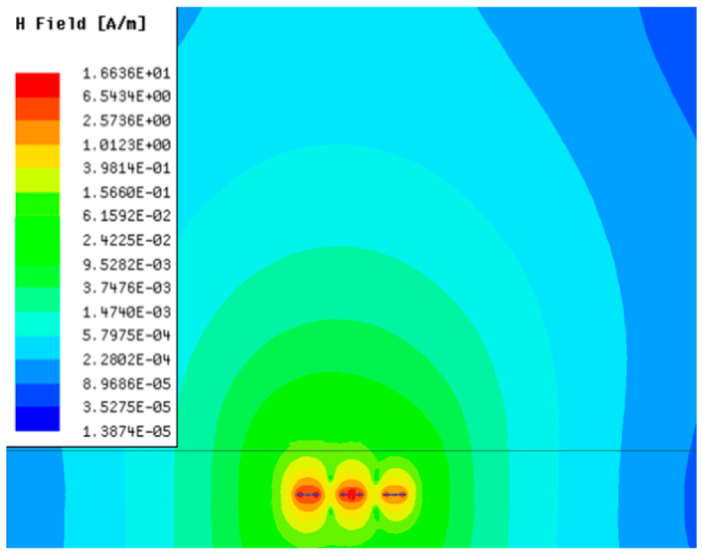
60°	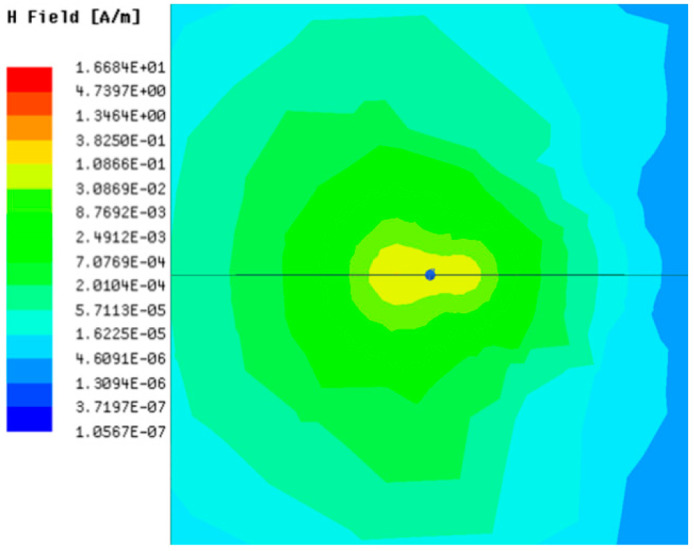	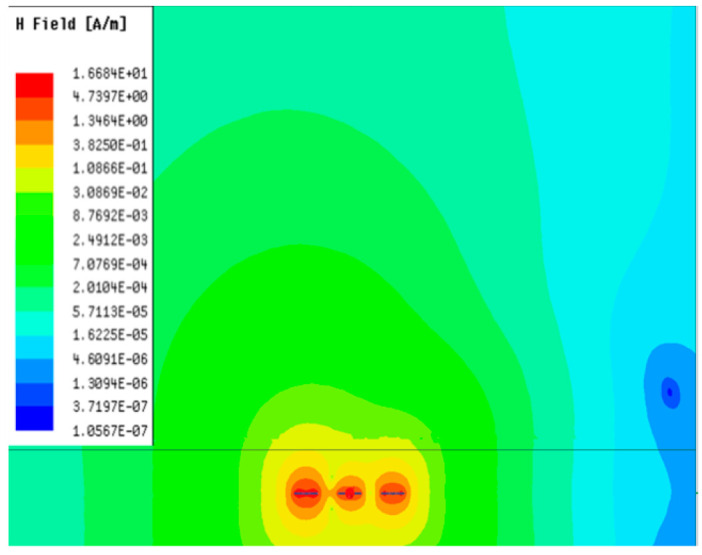
90°	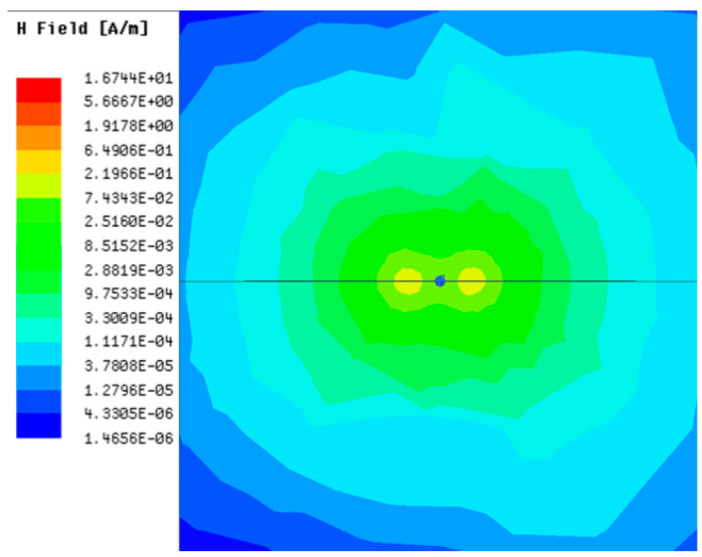	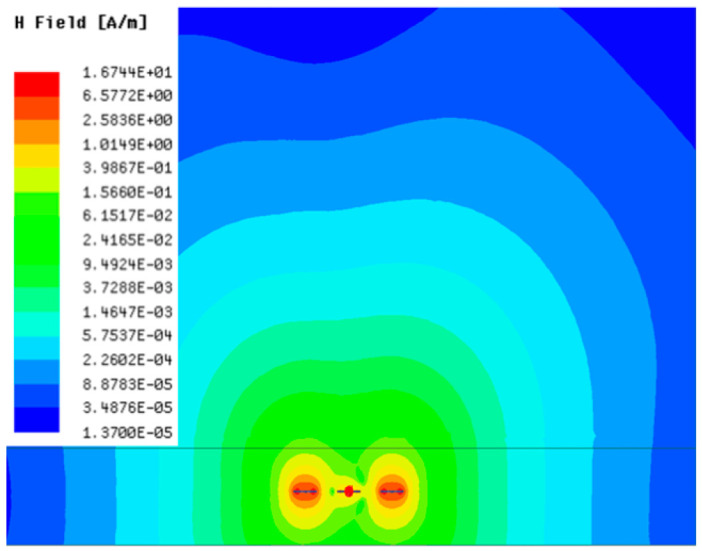

**Table 2:** Simulated magnetic field distribution of the beam-steering MIMO-WPT implemented with five TX coils.

Phase Step (*ψ*)	Azimuth (*xy*-Plane)	Elevation (*yz*-Plane)
0°	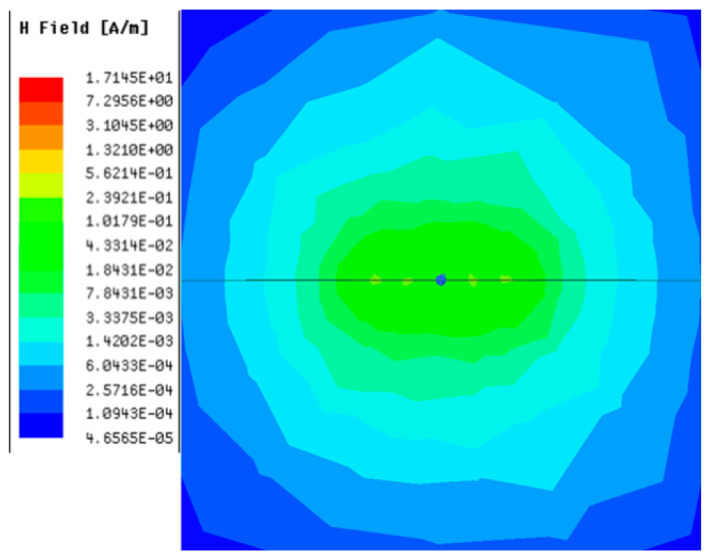	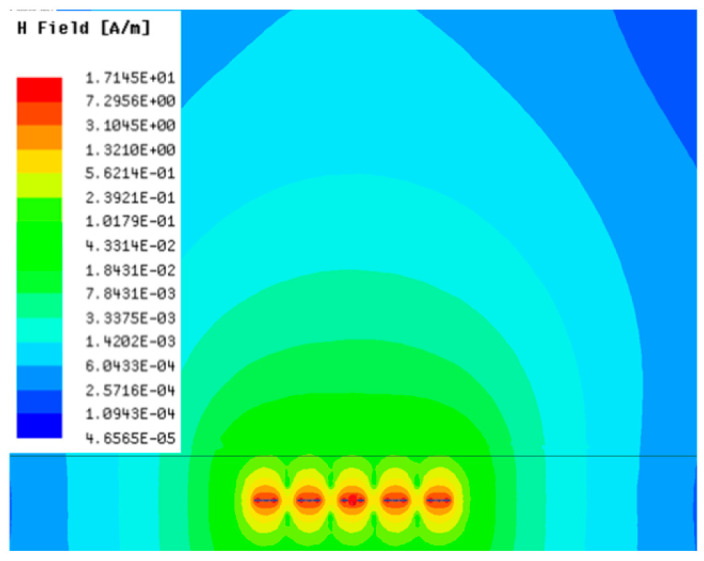
30°	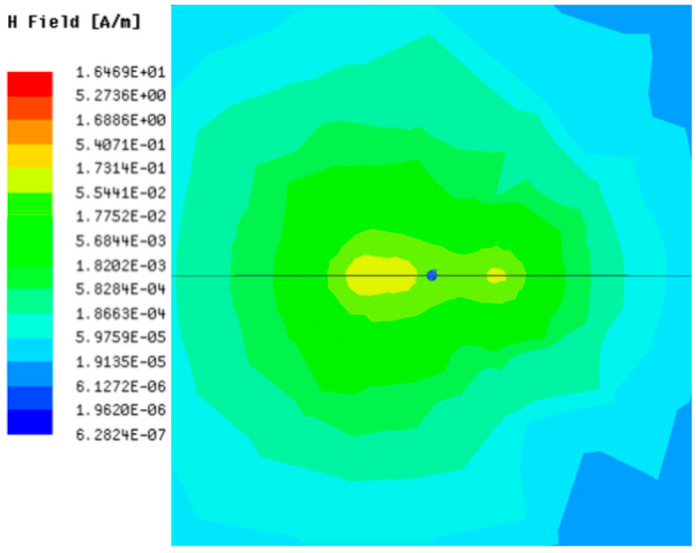	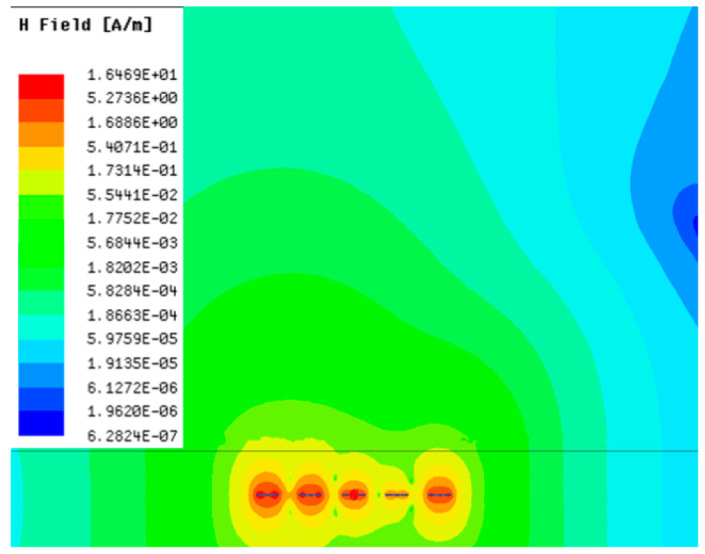
60°	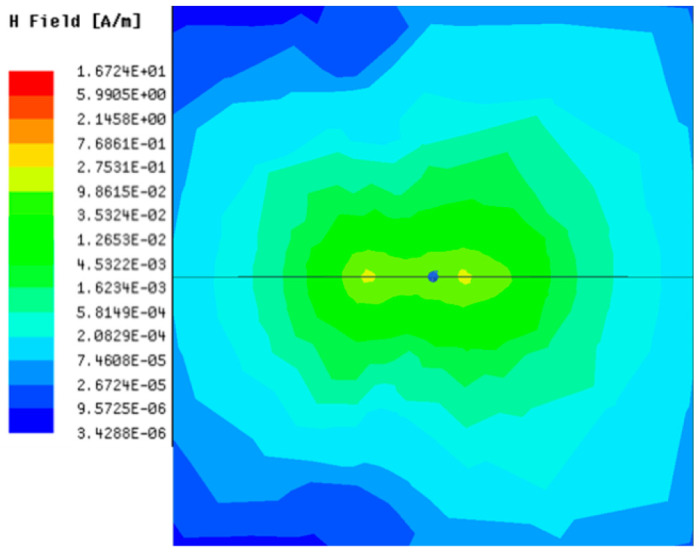	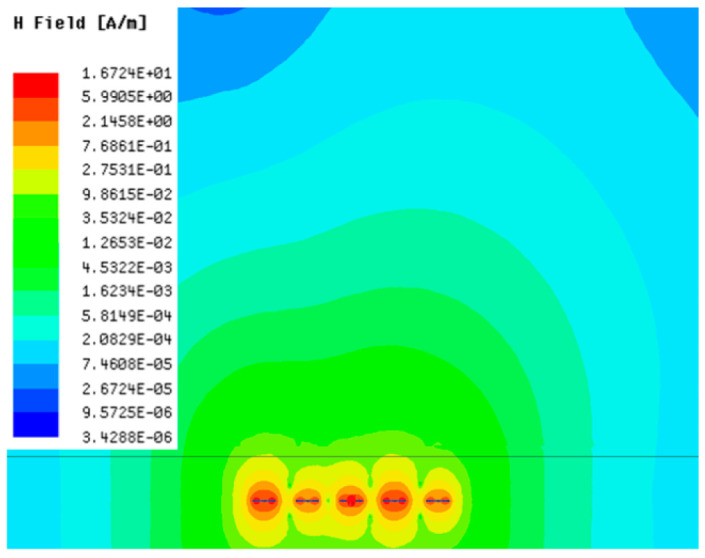
90°	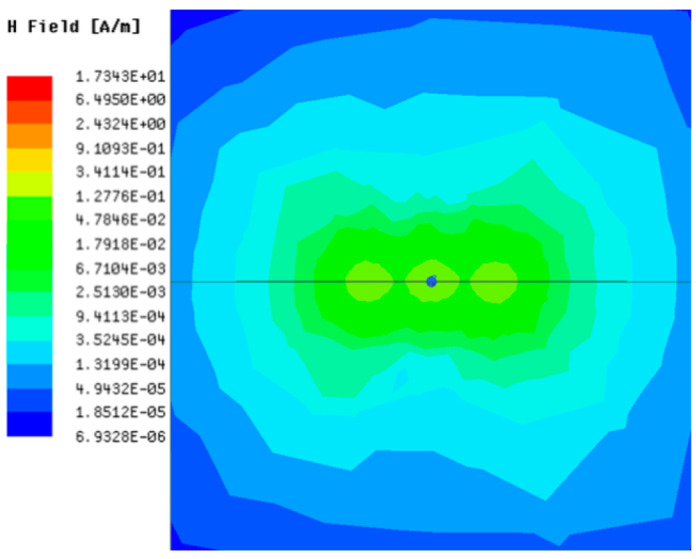	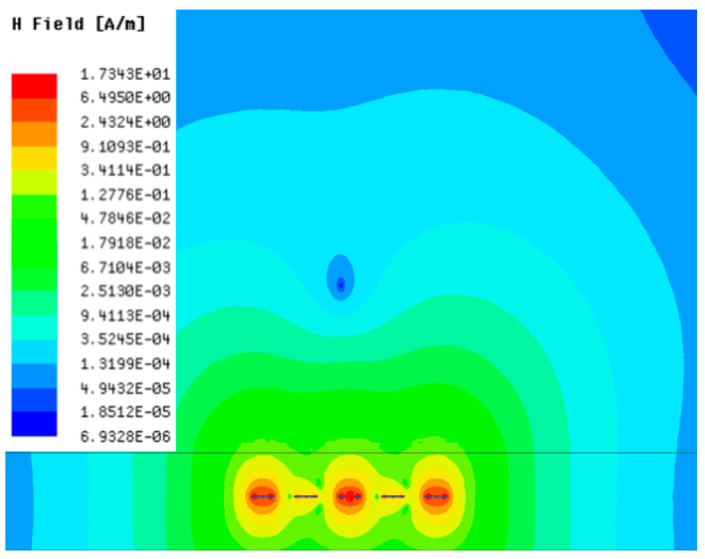

**Table 3:** Simulated magnetic field distribution of the beam-steering MIMO-WPT implemented with seven TX coils.

Phase Step (*ψ*)	Azimuth (*xy*-Plane)	Elevation (*yz*-Plane)
0°	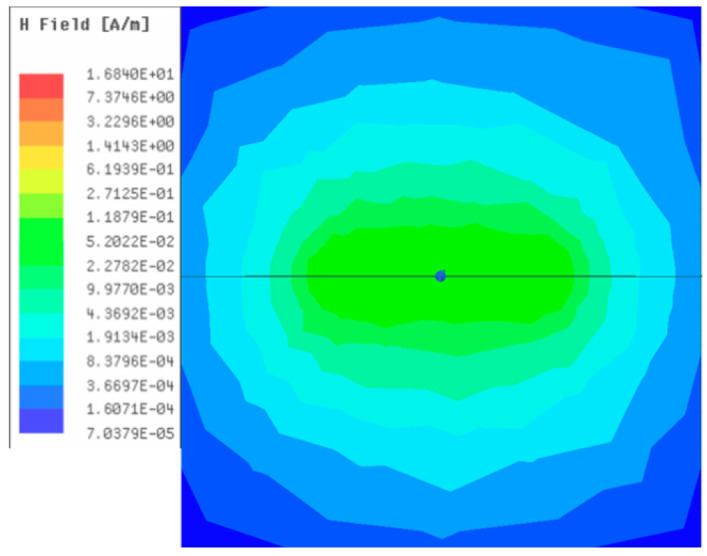	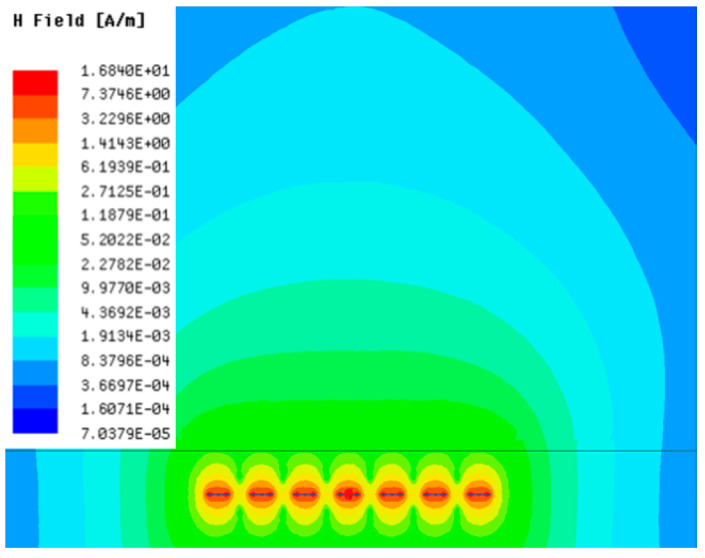
30°	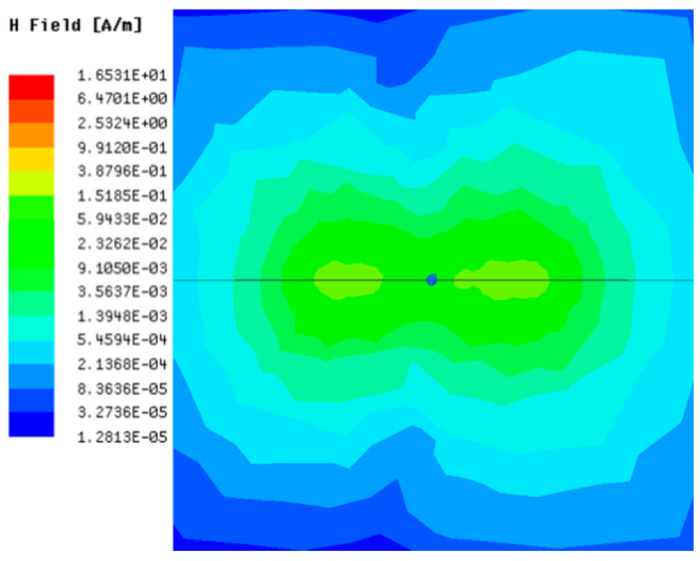	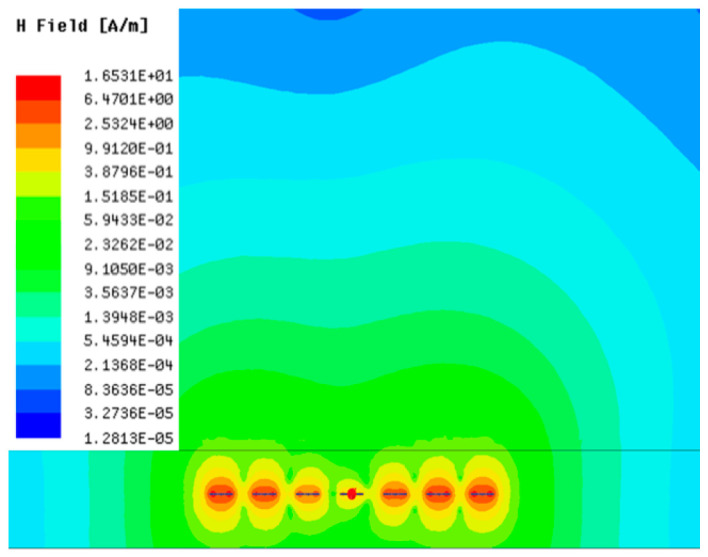
60°	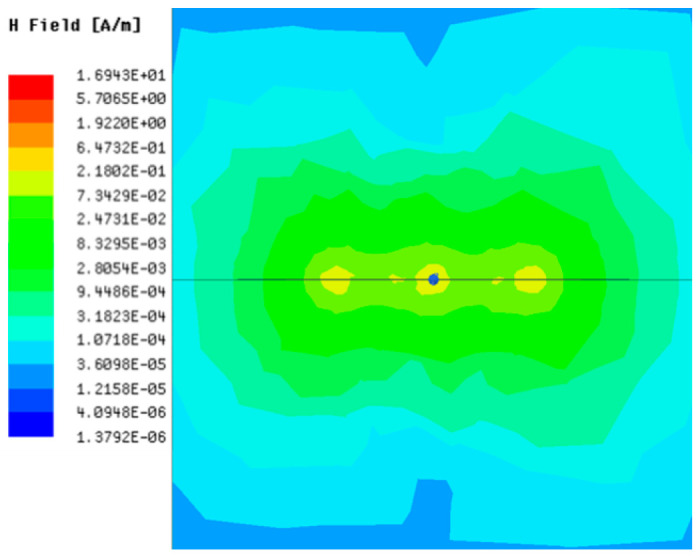	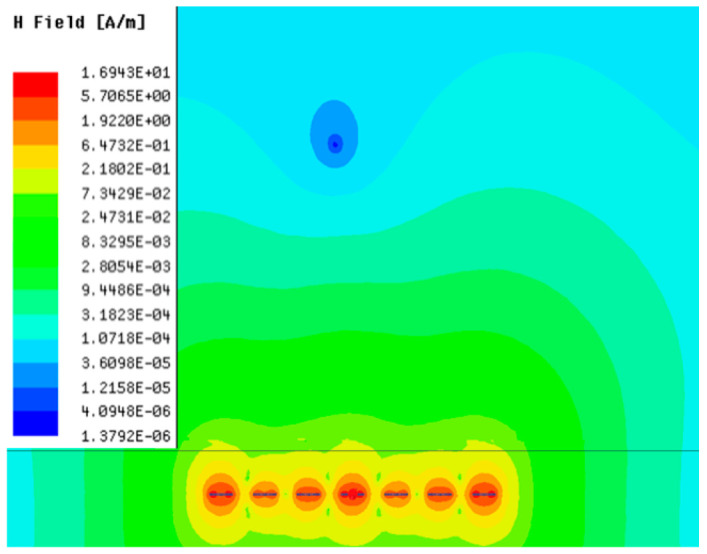
90°	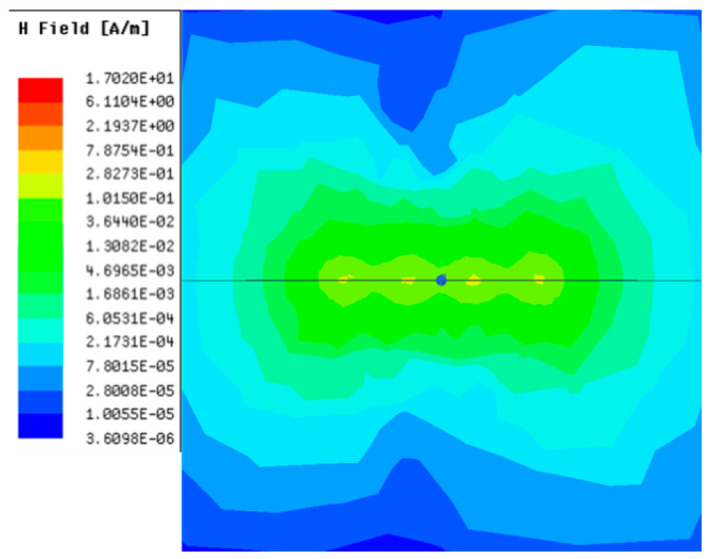	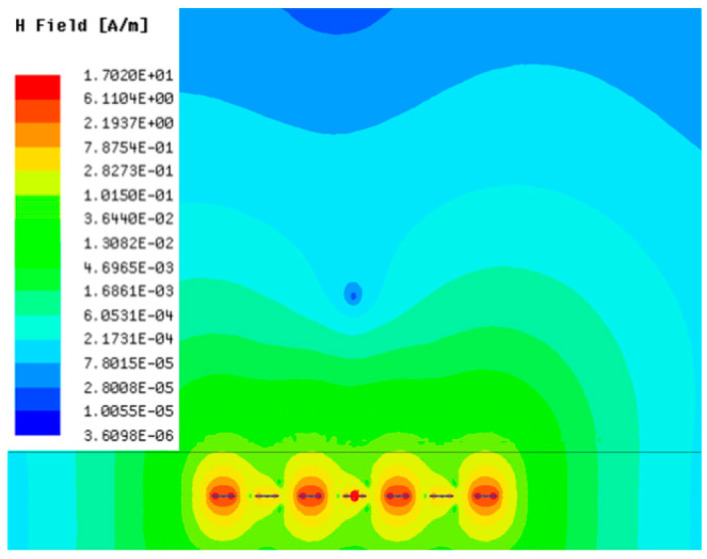

## Data Availability

Not applicable.
